# Successful Case of Ligature of the Right Subclavian Artery, for Axillary Aneurism

**Published:** 1827-06

**Authors:** 

**Affiliations:** Surgeon to the Artillery Hospital, Petersburgh.


*Successful Case of Ligature of the Right Subclavian Artery, for Axillary Aneurism.* By Dr. Arendt, Surgeon to the Artillery Hospital, Petersburgh. (Communicated by Robert Lee, M.D.)

When at Petersburgh, in the month of June last, I was invited by Dr. Arendt, chief surgeon of the Artillery Hospital, to be present at the operation of tying the right subclavian artery for axillary aneurism. The patient was about thirty years of age, a clerk in one of the government offices, and had enjoyed very good health prior to the period when the pulsating tumor appeared in the right axilla, and which was about the beginning of the month of May. The disease was not attributed to any violence inflicted upon the arm, but appeared to be of spontaneous origin.

The operation was performed by Dr. Arendt on the 6th of June, 1826, in the presence of nearly all the distinguished professional gentlemen of the Russian metropolis; and it excited much general attention, from being only the second case where the operation of tying the subclavian artery had been attempted in the empire. When the young man was laid upon the table, and properly supported by pillows, an opportunity was afforded me of examining the state of the axilla and arm. There was a strong pulsation in the armpit, under the pectoral muscle, and behind and above the clavicle; and to such an extent did it exist in this latter situation, that the greater number present suspected that the subclavian artery was involved in the disease. The arm was not swollen, and the pulsations of the radial artery could be felt at the wrist, though weaker than in the left side. The patient complained of pain in the aneurism, and of a disagreeable numbness and tingling sensation in the whole extremity; and his sleep for some time had been much disturbed by frightful dreams, and strong throbbings of the arteries of the head.

The incision in the integuments was begun about three-quarters of an inch from the sternal extremity of the clavicle, and carried about three inches outward parallel with and closely along the upper edge of that bone. The platysma-myoides, and subjacent cellular membrane, were divided by means of a director and scalpel; and the remaining parts of the operation were effected by the point of the aneurism-needle and the forefinger of the operator alone. When the connexions of the artery with the surrounding parts had been separated, its pulsations could be felt at the point where it issued from behind the scalenus anticus muscle. The depth of the wound was so great, and the opening so narrow and confined, that it seemed utterly impracticable by any means to pass a ligature around it. Many long and violent attempts were made to accomplish this with a strong needle, similar in principle and form to the *aiguille a ressort* of Desault, and the more recently invented needle of Mr. Bremner, but without success. At one time, when the artery was supposed to be over the bend of the needle, it was found that the pulsations in the axilla were not arrested by compression of this cord, which was supposed to be a mesh of the fibres of the scalenus anticus muscle; and consequently further attempts were required to inclose the vessel. At last the needle was actually passed behind the artery, and, when the ligature was tied around it, the pulsations of the aneurism and radial artery immediately ceased, and a sensation of numbness was at the same time experienced in the arm.

During the operation, the hemorrhage was very trifling indeed; not a vessel was cut which required to be tied. The patient was upon the table an hour and ten minutes. *The ligature was as thick as common whip-cord,* and the two ends, after it had been firmly tied round the artery, were left hanging out of the wound. The edges of the wound were brought together by slips of adhesive plaster, and covered with charpie.

On the sixteenth day after the operation, the ligature came away, and the pulsations in the axilla had entirely disappeared. Except a slight febrile attack on the second day after the operation, for which he was bled twice, no disagreeable symptom arose during the after treatment; and in four weeks the wound was completely closed, and the perfect use of the arm regained.

Dr. Gibbs was the first surgeon at Petersburgh who attempted to apply a ligature round the subclavian artery, and he has related a successful case in the Transactions of the Medico-Chirurgical Society of London. The other great arteries have likewise repeatedly been tied with success in Russia. Out of eight cases of ligature of the external iliac for aneurism, five of the patients were restored to perfect health. The carotid has only been tied once, and the result was fortunate. The femoral has been tied upwards of twenty times, and a large proportion of the cases has ended in the complete recovery of the patients.

2, *Upper John-street, Golden square;*
*April* 23*d*, 1826.

